# Clinical presentation and survival of childhood hypertrophic cardiomyopathy: a retrospective study in United Kingdom

**DOI:** 10.1093/eurheartj/ehy798

**Published:** 2018-12-06

**Authors:** Gabrielle Norrish, Ella Field, Karen Mcleod, Maria Ilina, Graham Stuart, Vinay Bhole, Orhan Uzun, Elspeth Brown, Piers E F Daubeney, Amrit Lota, Katie Linter, Sujeev Mathur, Tara Bharucha, Khoon Li Kok, Satish Adwani, Caroline B Jones, Zdenka Reinhardt, Juan Pablo Kaski

**Affiliations:** 1 Centre for Inherited Cardiovascular Diseases, Great Ormond Street Hospital, Great Ormond Street, London, UK; 2 Department of Paediatric Cardiology, Institute of Cardiovascular Sciences University College London, UK; 3 Department of Paediatric Cardiology, Royal Hospital for Children, Glasgow, UK; 4 Department of Paediatric Cardiology, University Hospitals Bristol NHS Foundation Trust, UK; 5 Department of Paediatric Cardiology, Birmingham Women and Children’s NHS Foundation Trust, UK; 6 Department of Paediatric Cardiology, University Hospital of Wales, Cardiff, UK; 7 Department of Paediatric Cardiology, Leeds Teaching Hospital NHS Trust, UK; 8 Department of Paediatric Cardiology, Royal Brompton Hospital and National Heart and Lung Institute, Imperial College London Harefield, UK; 9 Department of Paediatric Cardiology, University Hospitals of Leicester, UK; 10 Department of Paediatric Cardiology, Evelina London Children’s Hospital and Guys and St Thomas’ NHS Foundation Trust, UK; 11 Department of Paediatric Cardiology, University Hospital Southampton NHS Foundation Trust, UK; 12 Department of Paediatric Cardiology, Oxford University Hospitals NHS Foundation Trust, UK; 13 Department of Paediatric Cardiology, Alder Hey Children’s Hospital, Liverpool, UK; 14 Department of Paediatric Cardiology, The Freeman Hospital, Newcastle, UK

**Keywords:** Hypertrophic cardiomyopathy, United Kingdom, Survival, Aetiology

## Abstract

**Aims:**

Understanding the spectrum of disease, symptom burden and natural history are essential for the management of children with hypertrophic cardiomyopathy (HCM). The effect of changing screening practices over time has not previously been studied. This study describes the clinical characteristics and outcomes of childhood HCM over four decades in a well-characterized United Kingdom cohort.

**Methods and results:**

Six hundred and eighty-seven patients with HCM presented at a median age of 5.2 years (range 0–16). Aetiology was: non-syndromic (*n* = 433, 63%), RASopathy (*n* = 126, 18.3%), Friedreich’s ataxia (*n* = 59, 8.6%) or inborn errors of metabolism (IEM) (*n* = 64, 9%). In infants (*n* = 159, 23%) underlying aetiology was more commonly a RASopathy (42% vs. 11.2%, *P* < 0.0001) or IEM (18.9% vs. 6.4% *P* < 0.0001). In those with familial disease, median age of presentation was higher (11 years vs. 6 years, *P* < 0.0001), 141 (58%) presented <12 years. Freedom from death or transplantation was 90.6% (87.9–92.7%) at 5 years (1.5 per 100 patient years) with no era effect. Mortality was most frequently sudden cardiac death (SCD) (*n* = 20, 2.9%). Children diagnosed during infancy or with an IEM had a worse prognosis (5-year survival 80.5% or 66.4%). Arrhythmic events occurred at a rate of 1.2 per 100 patient years and were more likely in non-syndromic patients (*n* = 51, 88%).

**Conclusion:**

This national study describes a heterogeneous disease whose outcomes depend on the age of presentation and aetiology. Overall mortality and SCD rates have not changed over time, but they remain higher than in adults with HCM, with events occurring in syndromic and non-syndromic patients.

**Figure:**
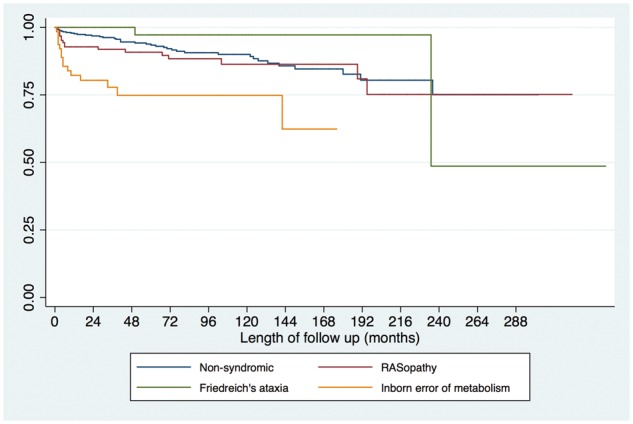

**See page 994 for the editorial comment on this article (doi: 10.1093/eurheartj/ehy889)**

## Introduction

Hypertrophic cardiomyopathy (HCM) is the second commonest cardiomyopathy during childhood, with an estimated annual incidence of 0.24–0.47 per 100 000.^1–3^ The disease in most children is caused by mutations in the cardiac sarcomere protein genes,^4^ but the aetiology is more heterogeneous than that seen in adults, and includes inborn errors of metabolism (IEM), neuromuscular disorders, and malformation syndromes.^5–7^

Understanding the spectrum of disease, symptom burden and outcomes are essential for the management of children with HCM. North American^2^^,^^5^ and Australian^3^^,^^8^^,^^9^ registry studies have provided valuable information on the epidemiology, aetiology and survival for childhood HCM, as well as morphological descriptions of the clinical phenotype at presentation.^7^ Long-term prognosis appears to depend on the age of presentation and aetiology.^5–7^ However, the effect of changing screening practices on the age of presentation, aetiology, and survival in childhood HCM has not been assessed. A detailed understanding of disease-specific causes of mortality and the incidence of outcomes other than mortality would enable a more individualized approach to patient care. This study sought to describe the clinical characteristics and outcomes of childhood HCM over four decades in a well-characterized United Kingdom (UK) cohort.

## Methods

A retrospective, longitudinal multi-centre cohort of children diagnosed with HCM in the UK between 1980 and 2017 was formed. Data were available from 13 out of 14 paediatric cardiac centres.

### Eligibility criteria

Patients aged 16 years or younger meeting the diagnostic criteria for HCM were eligible. This included patients with phenocopies of sarcomeric HCM (e.g. RASopathies, IEM, or neuromuscular diseases). The diagnosis of HCM was made if left ventricular wall thickness was greater than two standard deviations above the body surface area-corrected population mean (≥Z score +2), which could not be solely explained by abnormal loading conditions.^10^ Eligible patients were identified by the principle investigator at each site using multiple sources, including medical databases and echocardiography log books.

### Data collection

A single researcher (G.N.) visited each site to assist with data collection and confirm diagnostic classification. Anonymised, non-invasive clinical data were collected, including: demographics; aetiology; symptoms; medical therapy; family history; resting and ambulatory electrocardiography (ECG); 2D, Doppler, and colour transthoracic echocardiography; and exercise testing. Data were collected from baseline evaluation and last clinical review.

Aetiology was classified as non-syndromic in the absence of a diagnosis of a RASopathy syndrome (Noonan or other malformation syndrome), neuromuscular disease (including Friedreich’s ataxia), or inborn error of metabolism. Patients with mutations in the cardiac sarcomere protein genes or in other HCM-causing genes were included in the non-syndromic group.

### Clinical investigations

Non-sustained ventricular tachycardia (NSVT) was defined as three or more consecutive ventricular beats at a rate of greater than 120 b.p.m. with a duration of less than 30 s on ambulatory ECG recordings.^11^ An abnormal blood pressure (BP) response to exercise was defined as the failure of systolic BP to rise by ≥20 mmHg (flat BP response) or a fall in BP during exercise (hypotensive BP response).^11^^,^^12^

Echocardiographic measurements were made according to current guidelines.^13^ Specifically, maximal left ventricular wall thickness (MLVWT) was measured in the parasternal long-axis or parasternal short-axis views (2D or M-Mode) at end diastole. Left atrial diameter measurements were made in the parasternal long-axis view (2D or M-Mode) at end systole. Left ventricular outflow tract (LVOT) obstruction was defined as an instantaneous peak Doppler LVOT pressure gradient ≥30 mmHg at rest.^11^ A haemodynamically significant gradient was considered to be an instantaneous peak Doppler gradient ≥50 mmHg.^14^

### Outcomes

The primary patient outcomes, taken from last clinic appointment, were: sudden cardiac death (SCD) as previously defined^15^ or equivalent event [appropriate implantable cardioverter-defibrillator (ICD) therapy, aborted cardiac arrest, or sustained ventricular tachycardia (VT)]; heart failure-related death; cardiac transplantation; or non-cardiac death. Outcomes were assessed by experienced cardiologists at each site. Patients were classified as lost to follow-up if last clinical review was more than 3 years ago. In order to investigate possible era effects, patients were grouped according to the decade in which they were first diagnosed (1980–1989; 1990–1999; 2000–2009; 2010–2017).

### Statistical analysis

Body surface area was calculated from height and weight.^16^ Maximal left ventricular wall thickness and left atrial diameter absolute measurements are expressed in millimetres and as Z scores relative to the distribution of measurements vs. body surface area in normal children.^17^ Normally distributed continuous variables are described as mean ± standard deviation with two or three group comparisons conducted using Student *t-*test and analysis of variance (ANOVA), respectively. Skewed data are described as median [interquartile range (IQR)] with two or three group comparisons performed using Wilcoxon rank sum and Kruskal–Wallis tests, respectively. The distributions of categorical variables were compared using the *χ*^2^ test or Fisher’s exact test. Two-sample proportion tests were performed to compare groups within variables of interest. To account for multiple comparisons, the Bonferroni correction was applied. A significance level of 0.05 was used for all comparisons.

Estimates of survival were obtained using the Kaplan–Meier product limit method. Endpoints were all-cause mortality or cardiac transplantation, and a composite outcome of a major arrhythmic event (SCD, aborted cardiac arrest, sustained VT, or appropriate ICD therapy). Group differences in survival were assessed using the log rank test. Statistical analysis was performed using StataCorp. 2015. Stata Statistical Software: Release 14. College Station, TX, USA: StataCorp LP.

### Ethics

Ethics committee approval was obtained at each participating site. The study complies with the principles of Good Clinical Practice and the Declaration of Helsinki. G.N. and J.P.K. had access to all data and final responsibility for submission of the manuscript. All investigators have agreed to the manuscript as written.

## Results

**Figure 1:**
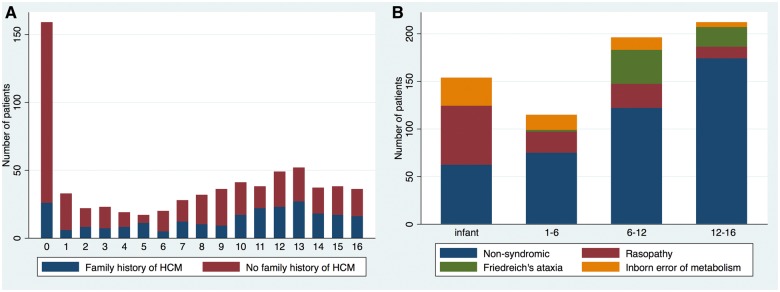
Age of presentation in years with childhood hypertrophic cardiomyopathy; (*A*) by family history, (*B*) by aetiology. Patients presenting in infancy are more likely to have a diagnosis of a RASopathy (*χ*^2^*P* < 0.001) or IEM (*χ*^2^*P* < 0.001).

Six hundred and eighty-seven patients diagnosed with HCM aged 16 years or younger were identified. Median age at presentation was 5.2 years (IQR 2.3–9.9 years; range 0–16 years). One hundred and fifty-nine patients (23%) presented under the age of 1 year and 314 (46%) during pre-adolescent years (1–12 years old) (*Figure 1A*). Four hundred and thirty-four patients (63%) were male.

### Aetiology

Data on aetiology are shown in *Table 1* and Supplementary material online, *Table S1.* Four hundred and thirty-three patients (63%) had non-syndromic HCM. Data on genetic testing strategy were not available. Pathogenic mutations in sarcomeric protein genes were reported in 100 (23%), most commonly in *MYH7* (*n* = 50) and *MYBPC3* (*n* = 39) (*Table 2*); twelve patients had more than one pathogenic mutation identified*.* One hundred and twenty-six patients (18.3%) had a RASopathy syndrome, 64 (9.3%) had an IEM and 59 (8.6%) Friedreich’s ataxia (Supplementary material online, *Table S2*). The number of patients with an underlying IEM or Friedreich’s ataxia increased over time (Supplementary material online, *Table S3*). Patients with Friedreich’s ataxia presented later in childhood [mean age of 10.4 years (range 4–16)], although four patients were diagnosed below the age of 7 years (*Figure 1B*).

**Table 1:** Clinical and demographic characteristics at diagnosis by age of diagnosis

		Whole cohort (no. of patients (%))	<1 year (no. of patients (%))	>1 year (no. of patients (%))	*P*-value
Gender	Male	434 (63.2%)	102 (64.2%)	332 (62.9%)	0.77*
Aetiology	Non-syndromic	433 (63%)	62 (39%)	371 (70.3%)	<0.0001
	Noonan syndrome (or RASopathies)	126 (18.3%)	67 (42%)	59 (11.2%)	<0.0001
	Friedreich’s ataxia	59 (8.6%)		59 (11.2%)	
	Other neuromuscular syndrome	5 (0.7%)		5 (1%)	
	Inborn error of metabolism	64 (9.3%)	30 (18.9%)	34 (6.4%)	<0.0001
Family history of HCM (*n* = 680)		242 (35.6%)	26 (16.4%)	216 (41.5%)	<0.0001*
Family history of SCD (*n* = 682)		50 (7.3%)	3 (1.9%)	47 (9%)	0.039*
NYHA/Ross at presentation (*n* = 684)	I	516 (75.4%)	94 (59.5%)	422 (80.2%)	<0.0001
	II	133 (19.4%)	42 (26.6%)	91 (17.3%)	0.12
	III	29 (4.2%)	17 (10.8%)	12 (2.3%)	<0.0001
	IV	6 (0.9%)	5 (3.2%)	1 (0.2%)	0.267
Cause of mortality (*n* = 75)	SCD	20 (2.9%)	2 (1.3%)	18 (3.4%)	
	CCF	12 (1.7%)	10 (6.3%)	2 (0.4%)	
	Other CV	12 (1.7%)	8 (5%)	4 (0.8%)	
	Non-CV	17 (2.5%)	12 (7.5%)	5 (0.9%)	
	Unknown	14 (2%)	3 (1.9%)	11 (2.1%)	

Data expressed as *n* (%). Total number of patients is 687 unless otherwise stated. All comparisons are made using a two-sample proportion test unless otherwise stated.

* Comparisons were made using χ^2^ test.

**Table 2:** Genes in which pathogenic mutations were identified in patients with non-syndromic hypertrophic cardiomyopathy

	Gene	Number of patients
Sarcomeric protein gene		100
	MYH7	50
	MYBPC3	39
	TPM1	4
	TNNT2	3
	TTN	2
	TNNI3	2
Non-sarcomeric gene		7
	DES	3
	PRKAG2	3
	JPH2	1
Variant of unknown significance (VUS)		10

### Family history

Two hundred and forty-two patients (35.6%) had a first-degree relative with HCM based on family history; of these, 214 (88%) had non-syndromic HCM. The median age of presentation was higher compared to those without a family history [11 years (5–13) vs. 6 years (0–12), *P* < 0.0001] and they were less likely to present during infancy (*Table 1* and *Figure 1A*). One hundred and forty-one patients (58%) with a family history presented in pre-adolescent years (<12 years). Fifty patients (7.3%) had a family history of SCD in a first-degree relative.

### Symptoms at presentation

Five hundred and sixteen patients (75.4%) were asymptomatic for heart failure symptoms at presentation [New York Heart Association (NYHA)/Ross functional Class 1]. Thirty-five patients (5.1%) had symptoms of congestive cardiac failure (CCF) (NYHA/Ross > 2). Heart failure symptoms were more common in patients presenting in infancy (*Table 1*)*.* Thirty-eight patients (5.6%) had a history of unexplained syncope at presentation. Twenty-four patients (3.5%) presented following an aborted SCD or out of hospital arrest.

### Investigations at baseline

Maximal left ventricular wall thickness on 2D echocardiography at baseline ranged from 4 to 48 mm (median 13 mm, IQR 11–18 mm, *n* = 645), with median Z score +10.1 (IQR +3.6 to +34.5). Left ventricular outflow tract gradient at baseline was documented in 604 (88%) patients; one hundred and sixty patients (26.5%) had LVOT obstruction and 32 patients (5.3%) had a gradient >90 mmHg (*Table 3*).

**Table 3:** Results of baseline investigations

			No. of patients (%)
Echocardiographic data at diagnosis (*n* = 677)	LVMWT (mm) [median (IQR)]		13 (11–18)
	LVMWT (Z score) [median (IQR)]		+10.1 (3.6–37)
	Left atrial diameter (mm) [mean (SD)] [*n* = 352]		29 (9.2)
	Left ventricular outflow gradient at rest (mmHg) [*n* = 604]	<30	444 (64.6%)
		30–50	47 (6.8%)
		50–90	81 (11.8%)
		>90	32 (12.1%)
Ambulatory ECG performed (*n* = 483)	Yes	NSVT	7 (1.4%)
		No NSVT	476 (98.6%)
	No		204 (29%)
Exercise test performed^a^ (*n* = 220)	Non-syndromic		196 (45%)
	RASopathy		12 (10%)
	Friedreich ataxia		7 (12%)
	Inborn error of metabolism		5 (8%)
Blood pressure response to exercise^a^	Normal (>20 mmHg rise in systolic BP)		126 (57%)
	Flat (<20 mmHg rise in systolic BP)		68 (31%)
	Hypotensive (fall in systolic BP)		16 (7%)

Data expressed as number (%). Total number of patients is 687 unless otherwise stated.

SD, standard deviation.

a Exercise test in patients ≥6 years.

Of 483 patients (71%) with 24-h ambulatory ECG at baseline, NSVT was detected in 7 (1.4%). An exercise test was performed in 220 out of 412 patients (53%) aged 6 years and above. Blood pressure response to exercise was classified as abnormal in 84 patients (38%), of which 23 (27%) had LVOT obstruction at rest and 37 (44%) were on beta-blocker therapy. No patient had documented arrhythmias during exercise.

### Outcomes

Median length of follow-up was 5.2 years (IQR 2.3–9.9 years). 529 (77%) patients remained under the age of 18 years at last follow-up. Ninety-eight patients (14.3%) were classified as lost to follow-up, of which 41 (42%) had been transitioned to adult care. one hundred and thirty-five patients (20%) underwent ICD implantation for primary (*n* = 108, 80%) or secondary (*n* = 27, 10%) prevention.

### Mortality

**Figure 2:**
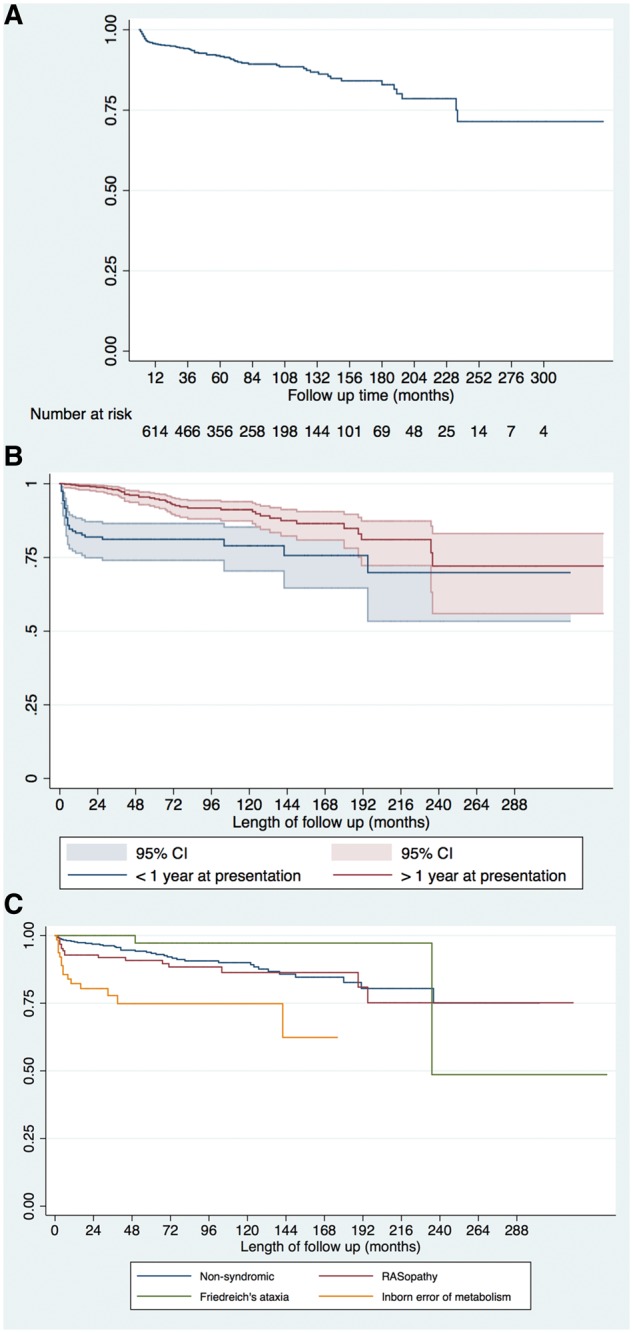
The Kaplan–Meier curves for survival free from all-cause mortality or cardiac transplantation; (*A*) for whole cohort; (*B*) stratified by age of presentation, Log rank test *P* < 0.0001. 95% CI are shown. (*C*) Stratified by aetiology, Log rank test <0.0001.

Six hundred and twelve patients (89.1%) were alive at last clinical follow-up. Seventy-five patients (10.9%) died: SCD in 20 (2.9%), non-cardiovascular (CV) in 17 (2.5%), CCF in 12 (1.8%), other CV causes in 12 (1.8%), and unknown in 14 (2.3%). Sixteen patients (80%) who died suddenly were aged under 18 years at the time of death. Ten patients (1.5%) underwent cardiac transplantation. Survival free from death or transplantation was 95.6% [95% confidence interval (95% CI) 93.7–96.9%] at 12 months and 86.3% (95% CI 82.6–89.2) at 10 years (*Figure 2A*). No difference in survival was seen by era of presentation (Supplementary material online, *Figure S1*). Children diagnosed during infancy or with an IEM had a worse prognosis, with survival at 12 months of 83.3% (95% CI 76.5–88.3%, *P* < 0.0001) or 82.2% (95% CI 70.2–89.8%) respectively (*Figure *2*B,C* and *Table 4*). Cause of death differed by underlying aetiology (Supplementary material online, *Table S1*) and age of diagnosis (*Table 1*).

**Table 4:** Survival rates from time of presentation to death or heart transplantation

		Survival after HCM diagnosis, % (95% confidence interval)			
		1 year	5 year	10 year	Log rank analysis
Whole cohort		95.6% (93.7–96.9)	90.6% (87.9–92.7)	86.3% (82.6–89.2)	
Age at presentation	<1 year (*n* = 159)	83.3% (76.5–88.3)	80.5% (73.2–85.9)	78.3% (69.7–84.7)	<0.0001
	>1 year (*n* = 528)	99.2% (97.9–99.7)	93.6% (90.6–95.6)	88.6% (84.3–91.7)	
Aetiology	Non-syndromic (*n* = 433)	97.6% (95.7–98.7)	92.7% (89.4–95)	87.5% (82.8–91.1)	<0.0001
	RASopathy (*n* = 126)	92.5% (86.1–96)	90.5% (83.4–95.6)	85.9% (76.7–91.7)	
	Inborn error of metabolism (*n* = 64)	82.2% (70.2–89.8)	66.4% (50.2–78.3)	66.4% (50.2–78.3)	
	Friedreich’s ataxia (*n* = 59)	100	97.1% (80.9–99.6)	97.1% (80.9–99.6)	

Survival rates, using the Kaplan–Meier method, with subgroup analysis based on age at diagnosis and aetiology. Patient numbers are indicated (*n*).

### Major arrhythmic events:

Fifty-eight patients (8.4%) had an arrhythmic event [SCD in 20 (34%); resuscitated cardiac arrest in 11 (19%); sustained VT in 8 (14%); appropriate ICD discharge in 19 (33%)] during follow-up, with an event rate of 1.2 per 100 patient years at risk. Freedom from arrhythmic event was 98% (95% CI 97.3–99.3) at 1 year, 92.9% (95% CI 90.3–94.9) at 5 years and 89.6% (95% CI 86.1–92.2%) at 10 years. Arrhythmic events occurred in 51 patients (88%) with non-syndromic HCM, 5 (9%) with a RASopathy, and 2 (3%) with an IEM (*Table 5*). No patients with neuromuscular disease (e.g. Friedreich’s ataxia) had an arrhythmic event. Nine patients (7.6%) who had been transitioned had arrhythmic events (SCD *n* = 4, appropriate ICD discharge *n* = 5).

**Table 5:** Clinical characteristic of patients with inborn error of metabolism or malformation syndrome and sudden cardiac death

	Diagnosis	Age at presentation	Symptoms at presentation	Phenotype	History of arrhythmias	Interventions	SCD or equivalent event
Patient 1	Danon’s disease	13 years	Unexplained syncope	Concentric LVMWT 30 mm LVOT 10 mmHg	Nil	ICD implanted aged 14 years	Sustained VT aged 16 years
Patient 2	Glycogen storage disease	3 months	NYHA 1	Concentric LVMWT 9 mm LVOT 6 mmHg	Nil	Reveal device implanted aged 14 (unexplained syncope aged 13)	SCD—reveal device documented VT + fine VF aged 14
Patient 3	Noonan’s	13 years	NYHA 2	ASH MWT 18 mm LVOT 58 mmHg	NSVT aged 16 years	Nil	SCD aged 18 years
Patient 4	Noonan’s	11 years	NYHA 1	Concentric LVMWT 18 mm LVOT 23 mmHg	NSVT aged 15 years	Primary prevention ICD implanted aged 16 years	Appropriate ICD therapy aged 16 years
Patient 5	Noonan’s	9 days	NYHA 2	BVH LVMWT 14 mm LVOT 32 mmHg	Nil	Myectomy aged 5 years	SCD aged 8 years
Patient 6	Other RASopathy	2 years	NYHA 1	ASH LVMWT 18 mm LVOT 16 mmHg	NSVT aged 9 years	Nil	Aborted cardiac arrest aged 10 years
Patient 7	Noonan’s	3 months	NYHA 3	BVH LVMWT 12 mm LVOT 73 mmHg RVOTO	Nil	Nil	Sustained VT aged 14 months

ASH, asymmetric hypertrophy; BVH, biventricular hypertrophy; LVMWT, left ventricular maximal wall thickness; RVOTO, right ventricular outflow tract obstruction; VF, ventricular fibrillatrion.

## Discussion

This study describes the clinical characteristics and outcomes of a national cohort of 687 childhood HCM patients, representing one of the largest studies in paediatric HCM, and the largest systematic study from Europe. Important novel findings include the detection of a phenotype in 58% of pre-adolescents with a first-degree family history of HCM and a change in the prevalence of syndromic HCM, particularly IEM, over time. Although the overall mortality and rates of SCD are lower than reported in early paediatric studies, they are nevertheless higher than seen in adults, where SCD rates are 0.81% per year. The cause of mortality differed by underlying aetiology, although arrhythmic events occurred in syndromic as well as non-syndromic patients.

### Comparison with previous studies

Current understanding of the aetiology and clinical characteristics of childhood HCM is primarily derived from a North American registry, which described 855 patients under the age of 18 years.^5^ The present study describes a cohort of patients under 16 years at diagnosis with a comparable length of follow-up [although recruited from a wider time period (1980–2017 vs. 1990–2009)], which provides a useful comparison of the clinical presentations and outcomes of childhood HCM in different healthcare systems. In agreement with the North American study, we report that the underlying aetiology of childhood HCM is heterogeneous; although the most common aetiology was non-syndromic, 37% had syndromic disease which was more common in infant-onset disease. More patients with an inborn error of metabolism were included in the most recent era (2010 onwards, *n* = 41) compared to earlier time periods (1990–1999, *n* = 1), which is likely to be due to systematic screening of patients with metabolic disease for cardiac disease. In the present study, only 23% of non-syndromic patients had a confirmed pathogenic mutation in a cardiac sarcomere protein gene. This is almost certainly an underestimate of the true prevalence as previous studies have shown that most cases of HCM are caused by sarcomeric mutations, even in young children.^4^

This study contains a higher number of non-syndromic patients with familial disease compared to the North American registry (*n* = 214, 49% vs. *n* = 109, 17%), of which over half were diagnosed with a phenotype in pre-adolescence. The age of presentation for familial disease is determined both by disease-specific and healthcare system factors, such as the age at which family screening started. Patients with more severe disease may therefore present at a younger age with symptoms whereas screening may identify children with a milder subclinical disease. It is not known if these patients were diagnosed through screening or in response to symptoms, but it is noteworthy that the majority were asymptomatic at presentation. Current guidelines recommend that routine family clinical screening is not started before the age of 10^11^ or 12 years,^18^ which may in part explain the peak in adolescence previously reported with patients’ first clinical assessment occurring in adolescent years. The age of presentation for familial disease is not described in other registry studies, but the findings from this study suggest that the age-related penetrance of HCM is highly variable and that consideration should be given to commencing screening at an earlier age.

### Outcomes of paediatric hypertrophic cardiomyopathy

This study shows that most children with HCM have a relatively good prognosis, with overall mortality or transplantation rates of 1.5 per 100 patient years at risk. Our data confirm previous reports^5^^,^^8^ that underlying aetiology has a significant impact on outcomes. Five-year survival was much lower for patients with an IEM (66%), compared to those with a diagnosis of a RASopathy syndrome (90.5%) or Friedreich’s ataxia (97%). These survival rates are higher than those previously reported in the North American registry,^5^ which likely reflects the variability in phenotype and outcomes that are seen. The cause of death differed depending on the underlying aetiology with non-cardiac deaths more common in patients with an IEM (*n* = 8, 12.5%) or RASopathy (*n* = 5, 4%). This highlights the multi-systemic nature of such conditions where cardiac involvement is only one aspect of the clinical phenotype and may not determine long-term outcomes. Also in agreement with previous reports,^5^^,^^8^ presentation at a young age was associated with worse outcomes. This finding may be partly explained by age-related difference in aetiology. However, it may also be a surrogate marker for disease severity as patients with more severe disease may be expected to present earlier in life and with worse outcomes. The phenotype and progression of familial hypertrophic cardiomyopathy in childhood, as well as the impact of clinical screening at a younger age, is largely unknown and warrants further investigation.

No era effect was seen on survival, although the earliest era (1980–1989) contained significantly smaller numbers of patients (Supplementary material online, *Table S3* and *Figure S1*), suggesting that the findings are an accurate reflection of the prognosis of childhood HCM. The explanation for this is likely multifactorial. The medical management of children with HCM has not significantly changed over the study period. However, ICDs have become widely used and shown to be effective at terminating malignant arrhythmias^19–21^ although at the expense of a higher incidence of complications compared to adult populations. Our data would support that ICDs are effective at terminating malignant arrhythmias as over time the proportion of patients experiencing SCD decreased whilst those with an aborted SCD/appropriate ICD therapy increased. Current guidelines recommend implantation of a primary prevention ICD in children with two or more risk factors for SCD.^21^ Nonetheless, challenges remain in identifying patients at highest risk for an arrhythmic event as evidenced by patients experiencing SCD/aborted arrests whilst under clinical follow-up and the low number of appropriate ICD therapies (14%). The higher number of infants with an inborn error of metabolism or RASopathy in recent years, who are known to have a worse prognosis, could also contribute to the lack of difference in survival seen between eras.

Arrhythmic events occurred at a rate of 1.2 per 100 patient years at risk. While this is significantly lower than initial reports,^22–24^ it remains higher than in adult cohorts (0.81% per year).^25^^,^^26^ Interestingly, seven of the patients who died suddenly had phenocopies of sarcomeric HCM, groups of patients that are traditionally regarded as having a low risk for an arrhythmic event. One of these patients had Danon syndrome, which is recognized to have a high risk of SCD,^27^ however five had a diagnosis of a RASopathy syndrome and one had a glycogen storage disorder. This highlights the need for a systematic assessment of arrhythmic risk in all patients with HCM, regardless of the underlying aetiology. It is noteworthy that there were no arrhythmic events in the 59 children with Friedreich’s ataxia, suggesting that patients with Friedreich’s ataxia may be at lower risk of ventricular arrhythmias. The finding that arrhythmic events occurred after transition to adult care confirms recent reports^28^ that arrhythmic risk in this group of patients is not limited to childhood and adolescence meaning ongoing surveillance is required.

### Limitations

This study is limited by inherent problems of retrospective studies, in particular missing or incomplete data. As no prospective database exists in the UK, all patients diagnosed with HCM over the study period (1980–2016) may not have been included. The number of patients by calendar year of diagnosis increased over time (Supplementary material online, *Table S3*). This may be due to more complete identification of affected patients. However, it may also represent a change in clinical practice, with earlier screening of affected families and routine cardiac assessment for patients with multi-systemic syndromes or diagnoses. Fourteen percentage of patients were classified as lost to follow-up (last clinical review more than 3 years ago) of whom 42% had been transitioned to adult care. Although the mortality rate is unlikely to be affected by this missing data due to nationally recorded death data in the UK National Health Service (NHS), other outcomes, such as arrhythmic events, could be underestimated. As patients were recruited from a number of centres, variations in clinical assessment and management between sites are inevitable. However, this is also a strength of the study, as it reflects accurately the clinical management of patients in the UK, both historically and in the current era.

**Take home figure:**
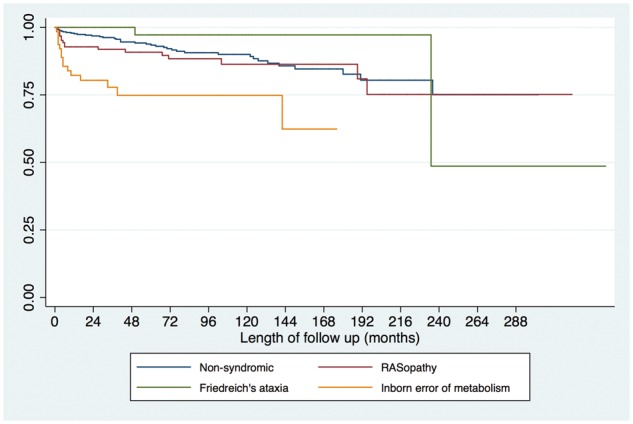
Kaplan-Meier curves for survival free from all cause mortality or cardiac transplantation. Aetiology is important for outcome with patients with an inborn error of metabolism having a worse prognosis most commonly secondary to congestive cardiac failure (log rank test <0.0001).

## Conclusions

This study is the first European multi-centre investigation of paediatric HCM. The results show that, during childhood, HCM is a heterogeneous disease in terms of its age of presentation, aetiology, and outcomes. Important novel findings include the detection of a phenotype in pre-adolescents with a family history of HCM and a change in the prevalence of syndromic HCM, particularly IEM, over time. Although in most children survival is good (freedom from death or transplantation was 90.6% at 5 years), SCD is the most common cause of mortality, occurring at a rate of 1.2 per 100 patient years at risk. Patients diagnosed in infancy or with an inborn error of metabolism have a worse prognosis, most commonly secondary to CCF or non-CV death, but arrhythmic events are also seen in these patient groups. An improved understanding of the relationship between aetiology, phenotype, and outcomes will facilitate the systematic study of risk factors for adverse events in this diverse patient group.

## Supplementary Material

Supplementary Figure 1

Supplementary Table 1

Supplementary Table 2

Supplementary Table 3
