# Supplementary figures and images for: Clinical presentation and survival of childhood hypertrophic cardiomyopathy: a retrospective study in United Kingdom
Source: Eur Heart J. 2018 Dec 6;40(12):986–93. doi: 10.1093/eurheartj/ehy798 (PMC6427088; doi:10.1093/eurheartj/ehy798)

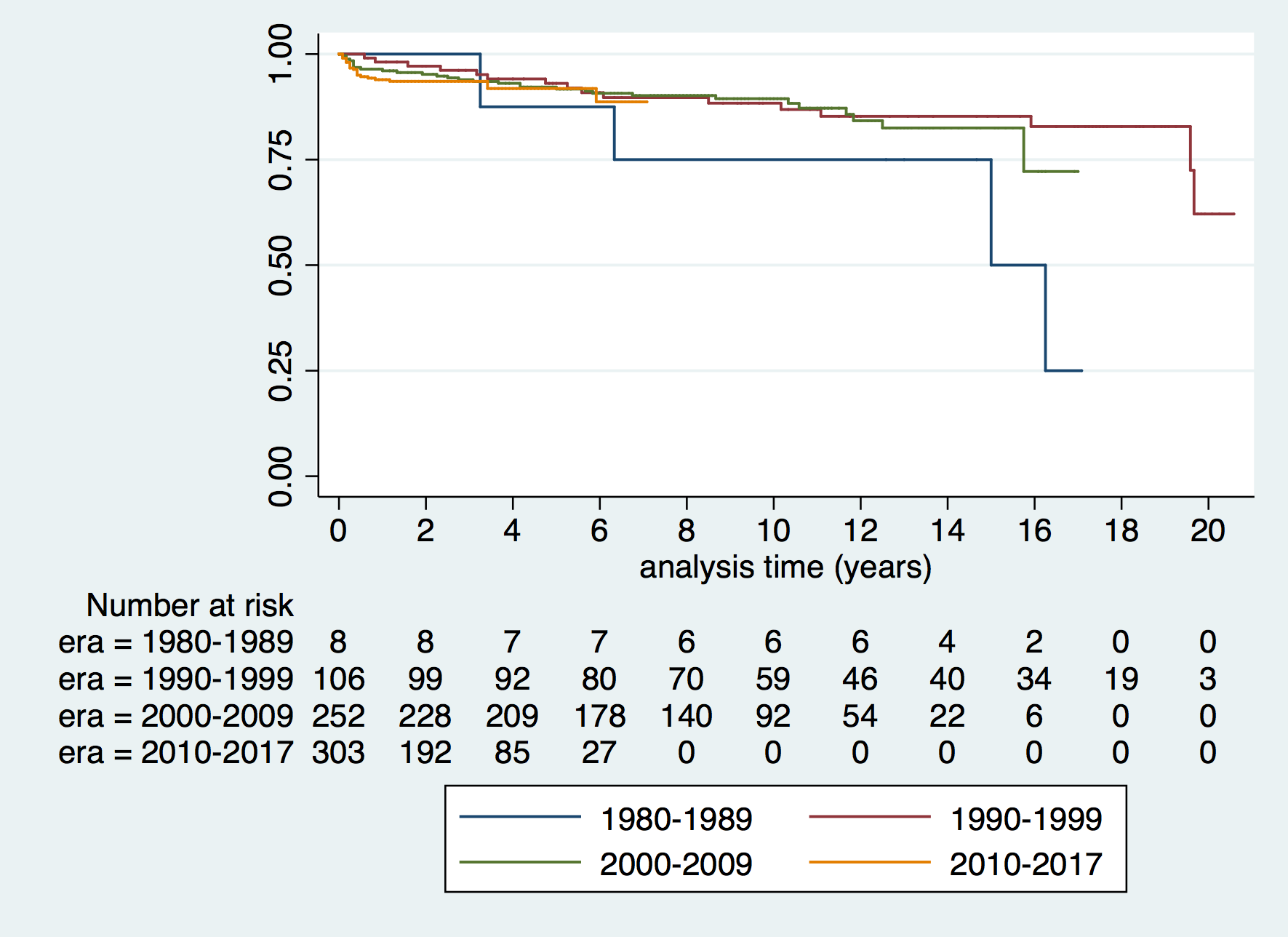

Supplement: Supplementary Figure 1 [file ehy798_supplementary_figure_1.png]
