# Supplementary material for: Clinical presentation and survival of childhood hypertrophic cardiomyopathy: a retrospective study in United Kingdom
Source: Eur Heart J. 2018 Dec 6;40(12):986–93. doi: 10.1093/eurheartj/ehy798 (PMC6427088; doi:10.1093/eurheartj/ehy798)
Supplement: Supplementary Table 1 [file ehy798_supplementary_table_1.docx]

**Supplementary material**

**Supplementary Table 1: Clinical and demographic characteristics by diagnosis**

|  |  | | Non-syndromic (n=433) | Noonan syndrome (or RASopathies) (n=126) | Friedreich’s ataxia  (n=59) | Inborn error of metabolism  (n=64) |
| --- | --- | --- | --- | --- | --- | --- |
| Gender | Male | | 286 (66%) | 83 (66%) | 28 (47%) | 33 (52%) |
| Age at presentation (years) | | <1 | 62 (14%) | 67 (53%) | 0 | 30 (47) |
|  |  | 1-5 | 75 (17%) | 22 (17%) | 2 (3%) | 16 (25) |
|  |  | 6-11 | 122 (28%) | 25 (20%) | 36 (61%) | 13 (20%) |
|  |  | 12-16 | 174 (40%) | 12 (10%) | 21 (36%) | 5 (8%) |
| Family history of HCM  (n = 680) | | | 214 (50%) | 8 (6%) | 9 (15%) | 11 (17%) |
| Family history of SCD  (n = 682) | | | 45 (10.5%) | 1 (1%) | 2 (4%) | 2 (3%) |
| NYHA/Ross at presentation (n = 684) | I | | 345 (80%) | 77 (61%) | 48 (83%) | 41 (65%) |
|  | II | | 71 (16%) | 41 (33%) | 9 (15%) | 12 (19%) |
|  | III | | 13 (3%) | 7 (6%) | 0 | 9 (14%) |
|  | IV | | 3 (1%) | 1 (<1%) | 1 (2%) | 1 (2%) |
| **Cause of mortality (n=75)** | SCD | | 18 (4.2%) | 2 (1.6%) | 0 | 0 |
|  | CCF | | 4 (1%) | 4 (3.2%) | 0 | 4 (6.3%) |
|  | Other CV | | 7 (1.6%) | 4 (3.2%) | 0 | 1 (1.6%) |
|  | Non-CV | | 3 (0.7%) | 5 (4%) | 1 (1.7%) | 8 (12.5%) |
|  | Unknown | | 8 (1.8%) | 2 (1.6%) | 0 | 3 (6.3%) |

Data expressed as number (%). Total number of patients is 687 unless otherwise stated. CCF = congestive cardiac failure, CV = cardiovascular
