# Supplementary material for: Clinical presentation and survival of childhood hypertrophic cardiomyopathy: a retrospective study in United Kingdom
Source: Eur Heart J. 2018 Dec 6;40(12):986–93. doi: 10.1093/eurheartj/ehy798 (PMC6427088; doi:10.1093/eurheartj/ehy798)
Supplement: Supplementary Table 2 [file ehy798_supplementary_table_2.docx]

**Supplementary table 2: Diagnoses of patients with an inborn error of metabolism, neuromuscular or malformation syndrome.**

|  |  |  | Number of patients |
| --- | --- | --- | --- |
| Inborn error of metabolism (n=64) | Storage disorders | GSD III | 9 |
|  |  | GSD IIb (Danon disease) | 6 |
|  |  | GSD II (Pompe disease) | 9 |
|  |  | Mucopolysaccharide storage disorder | 12 |
|  | Mitochondrial disorders | MELAS | 2 |
|  |  | Leigh syndrome | 1 |
|  |  | Sengers syndrome | 2 |
|  |  | Congenital lactic acidosis | 3 |
|  |  | Mitochondrial cytopathy | 7 |
|  |  | Mitochondrial respiratory chain disorders | 1 |
|  | Disorders of fatty acid metabolism | Carnitine transporter deficiency | 2 |
|  |  | VLCADD | 2 |
|  | Disorder of glycosylation | Carbohydrate glycoprotein deficiency | 2 |
|  | Disorder of amino acid metabolism | Glutaric acidemia type 3 | 1 |
|  | Other | Molybdenum cofactor deficiency | 2 |
|  |  | Purine nucleoside phosphorylase deficiency | 1 |
|  |  | Aicardi-goutieres syndrome | 1 |
|  |  | Kabuki Syndrome | 1 |
| Neuromuscular disease (n=64) | Friedreich's Ataxia | Friedreich’s Ataxia | 59 |
|  | Other neuromuscular disease (n=5) | Becker muscular dystrophy | 1 |
|  |  | Emery Dreifuss muscular dystrophy | 1 |
|  |  | Myofibrillar myopathy | 1 |
|  |  | Downs syndrome | 1 |
|  |  | Unknown | 1 |
| RASopathy (n= 126) | Noonan Syndrome | Noonan syndrome | 110 |
|  | Other RASopathy (n=16) | Costello syndrome | 5 |
|  |  | Noonan syndrome with multiple lentigines | 10 |
|  |  | Cardiofaciocutaneous syndrome | 1 |

GSD = Glycogen Storage Disorder, MELAS= Mitochondrial encephalomyopathy, lactic acidosis, and stroke-like episodes, VLCADD= Very long-chain acyl-coenzyme A dehydrogenase deficiency
