# Supplementary material for: Clinical presentation and survival of childhood hypertrophic cardiomyopathy: a retrospective study in United Kingdom
Source: Eur Heart J. 2018 Dec 6;40(12):986–93. doi: 10.1093/eurheartj/ehy798 (PMC6427088; doi:10.1093/eurheartj/ehy798)
Supplement: Supplementary Table 3 [file ehy798_supplementary_table_3.docx]

**Supplementary table 3: Clinical characteristics and survival rates by era of presentation.**

|  |  | 1980-1989  (n=11) | 1990-1999 (n=117) | 2000-2009 (n=254) | 2010-2017 (n=305) | P value |
| --- | --- | --- | --- | --- | --- | --- |
| Gender | Male | 6 (55%) | 67 (57%) | 161 (63%) | 200 (66%) | + 0.412 |
| Syndrome | Idiopathic/non-syndromic | 5 (45%) | 88 (75%) | 157 (62%) | 183 (60%) | + 0.025 |
|  | RASopathy | 4 (36%) | 19 (16%) | 48 (19%) | 55 (18%) |  |
|  | Friedreich ataxia | 1 (10%) | 9 (8%) | 26 (10%) | 23 (8%) |  |
|  | Inborn error of metabolism | 1 (10%) | 1 (1%) | 21 (8%) | 41 (13%) |  |
| Length of follow up in years (median, IQR) | | 15  (12.6-24) | 11.8  (6.5 - 18) | 8.5 (5.25-11.2) | 2.6 (1.3 - 4.1) |  |
| Mortality rate (per 100 person years at risk) | | 2.27  (0.852 - 6.05) | 1.38 (0.890-2.14) | 1.71 (0.123-2.373) | 2.54  (1.672 - 3.858) | * 0.735 |
| SCD rate (per 100 person years at risk) | | 1.13  (0.284 - 4.537) | 1.17  (0.729 - 1.886) | 1.14  (0.765-1.703) | 1.73  (1.044 - 2.872) | * 0.876 |

Data expressed as number (%). Total number of patients is 687 unless otherwise stated. + Indicates comparisons were made using Chi square test. * Indicates comparison made using Log Rank Test. SCD = sudden cardiac death
